# Peer‑led harm reduction in Brazil: gender‑based violence, intersectionality, and decolonial perspectives of community-based care

**DOI:** 10.1186/s12954-026-01499-7

**Published:** 2026-07-18

**Authors:** Delia Da Mosto, Ueslei Solaterrar

**Affiliations:** 1 Department of Global Health and Social Medicine, King’s College London, London, UK; 2 Instituto de Medicina Social, Universidade do Estado do Rio de Janeiro, Rio de Janeiro, RJ Brasil

**Keywords:** Harm reduction, Gender‑based violence, Trans care, Community mental health, Decolonial public health, Situated knowledges, Sex work

## Abstract

**Background:**

Gender-based violence (GBV) and substance use are closely linked in the lives of trans women and *travestis*. However, mainstream harm reduction frameworks rarely address these issues or focus on community-based practices that emerge in response. In Brazil, trans women and *travestis*—who are disproportionately Black, engaged in sex work and exposed to lethal violence—navigate health systems marked by transphobia, racism, and the dismantling of harm reduction policies. This study examines how GBV and substance use intersect in their lives, and the role of peer-led harm reduction practices as technologies of care and life-sustaining strategies.

**Methods:**

This article draws on two multisited ethnographic studies conducted in Brazil between 2018 and 2023. The first (2018–2020) examined suffering, self-care, and community-based support among trans women and *travestis* in Rio de Janeiro, combining life history and semi-structured interviews. The second (2022–2023) explored sex work, violence, resistance, and practices of care among cis and trans women sex workers, through participant observation, semi-structured interviews, and *rodas de conversa* (collective conversation circles). The two studies were placed in dialogue and analysed using thematic analysis supported by the Framework Method, through an intersectional and socio-anthropological lens.

**Results:**

Four interrelated themes emerged: (1) GBV was enacted across families, schools, health services, labour markets, and sex work spaces, producing cumulative trajectories of exclusion shaped by racism, cisnormativity, and class inequality; (2) substance use was inseparable from trauma, social suffering, and the labour conditions of sex work—embedded in an economy of survival rather than reducible to individual pathology; (3) despite structural exclusion, participants generated situated harm reduction practices—peer-led outreach, informal knowledge networks, collective care, and mutual protection—functioning as counter-hegemonic forms of health knowledge; (4) participants re-signified marginality as a site of agency, transforming exclusion into professional expertise, chosen kinship, and political activism.

**Conclusions:**

This study advances a conception of harm reduction that is intersectional, decolonial, and politically grounded. It demonstrates that GBV is not peripheral to substance use but constitutive of it, and that lived experience-generated knowledge, forged in structural oppression, is a legitimate foundation for harm reduction practices. These findings call for frameworks that move beyond individual risk reduction toward a social determination of health perspective—locating harm in intersecting systems of racism, transphobia, patriarchy, and criminalisation of sex work. Trans women and *travestis* must be recognised not only as populations exposed to harm, but as producers of care, knowledge, and political claims whose expertise is indispensable to advancing harm reduction theory and practice.

## Introduction

Harm reduction has long been recognised as a pragmatic and rights‑based response to substance use and its associated risks. Yet mainstream harm reduction frameworks, reflecting hegemonic health perspectives, have often been criticised for neglecting the ways gender, sexuality, race, and other intersecting inequalities shape experiences of harm [1, 2]. Gender‑based violence (GBV) can be both a driver and a consequence of substance use: women and LGBTIQA+ subjectivities who experience violence are more likely to use substances as coping mechanisms, while substance use can in turn increase exposure to further violence [3].

Intersectionality, originally [4], provides an essential framework for understanding these dynamics. Rather than treating gender, race, sexuality, or class as separate categories, an intersectional lens highlights how overlapping systems of oppression shape both vulnerabilities and forms of resilience [5]. As Ussher et al. [6] demonstrate in their study racism, transphobia, misogyny, class, and migration status intersect in what they describe as a “matrix of domination,” producing unique vulnerabilities that cannot be understood through a single axis of identity. Recent calls for intersectional harm reduction emphasise the need to address stigma, discrimination, and structural violence [7] in ways that move beyond biomedical models [8, 9].

A human rights perspective further expands harm reduction beyond the reduction of individual risk behaviours, situating it within broader processes of vulnerability reduction [10, 11]. From this perspective, drug-related harms are not produced by substance use alone, but also by the social, political, legal, and institutional conditions that expose some groups to violence, stigma, criminalisation, and barriers to care [10, 11]. Human rights violations may therefore contribute to harm both by creating the conditions in which risky practices become more likely and by limiting access to prevention, protection, and treatment [10]. This is particularly relevant for trans women and *travestis*^1^ engaged in sex work, whose experiences of substance use are shaped by intersecting forms of gendered, racialised, sexual, and territorial exclusion [11].

From a public health perspective, these vulnerabilities are not evenly distributed but are structured by broader systems of power. Hegemonic models rooted in capitalism, patriarchy, racism, and colonialism have historically defined humanity through the figure of the “first‑class citizen: European, male, rational, white, heterosexual, cisgender, and Christian” [12, 13]. Gonzaga [14] emphasises that racism—and, by extension, gender‑based violence—should not be treated merely as theoretical constructs but as fundamental ethical concerns within the civilizational framework of coloniality. This framework ordered the hierarchisation of people by skin colour [15] and by the anatomy of the genitalia [16]. Those with non‑conforming bodies and genders [17], or who engage in stigmatised sexualities and sex work [18], are relegated to zones of abjection, where their intelligibility is constantly questioned [19]. Yet, as hooks [20] reminds us, marginality is not only a site of exclusion but also a potential space of resistance and radical possibility.

Brazil's colonial history—marked by more than 350 years of enslavement of Indigenous and Black people—has produced a deeply racialised social structure in which anti-Black and anti-Indigenous racism operate as structural and institutional forces [21, 22]. National identity was forged not through reparation but under the myth of racial democracy and scientific racism [23–27]. Today, Black and mixed-race people constitute 56.7% of the population, yet they face persistent inequalities in access to housing, health, education, and security, while fighting for their land and against racial prejudice [28–31]. While legislative advances—including the Statute of Racial Equality (2010) and the National Policy for Comprehensive Health of the Black Population (2009)—represent important gains, structural inequalities remain deeply entrenched, rooted in historical processes that no single policy has been able to dismantle.

These colonial legacies are acutely impacting those at the intersection of race, gender and sexuality. In Brazil, gender-based violence against Indigenous women and girls increased by 258% between 2014 and 2023 [32], and the picture is even more alarming for Black trans women and *travestis* [33]. Similarly, sex workers, trans women and *travesti* are disproportionately exposed to GBV, social exclusion, and substance‑related harms. According to the 2025 report of the *Associação Nacional de Travestis e Transexuais*, at least 60% of murders of trans people worldwide occur in Brazil, with 78% of victims being Black* travesti* and trans women, and over 90% engaged in sex work [34]. Multiple studies further show that healthcare services often reproduce broader social inequalities, limiting access for sex workers and trans people despite policy reforms [35–40].

In Brazil, harm reduction has a distinct political history, inseparable from the Psychiatric Reform movement and the construction of the SUS (*Serviço Único de Saúde—Brazilian National Health System)*. Beyond a set of clinical techniques, harm reduction has been theorised and practised as a clinical-political paradigm—an ethics of care grounded in autonomy, territorial care, social inclusion, human rights and citizenship [41–43]. The 2006 Drug Law (*Lei 11.343/2006*) enshrined harm reduction as the primary framework for responding to substance use among people who cannot or do not wish to stop. However, from 2015 onwards, and particularly under far‑right governments (2018–2022), this framework was systematically dismantled: the 2019 Drug Law (*Lei 13.840/2019*) replaced harm reduction with abstinence as the central goal, redirected public funding toward Therapeutic Communities—largely religious, asylum-like institutions—and intensified a punitive approach to drug use. These shifts disproportionately affected already-marginalised populations, including trans women, *travestis*, and sex workers, whose access to community-based care was further eroded.

Within this context, Brazilian trans and *travesti* communities, together with workers and activists in the anti‑asylum movement, have developed situated practices of solidarity and harm reduction. These include peer‑led outreach in sex work zones, informal networks of knowledge sharing, and community self‑care initiatives. Such practices resist biomedical hegemony and generate new forms of autonomy, emerging as vital strategies for survival and resistance [40, 44, 45].

This article contributes to these debates by examining how GBV and substance use intersect in the lives of trans women^2^ and *travesti* in Brazil, and how community‑based formal and informal harm reduction practices emerge in response. It analyses their role as technologies of care and life sustenance. By centering lived experiences, it argues for a broader, politically grounded undestanding of harm reduction as a practice of care and resistance, forged from the margins and irreducible to biomedical intervention.

## Methodology

### Study design

This article draws on two qualitative ethnographic studies conducted in Brazil between 2018 and 2023. The first study examined the management of suffering, self-care, and community-based support among trans women and *travestis* whose lives and livelihoods were rooted in, or passed through, Rio de Janeiro's street-based context in Rio de Janeiro. The second study (carried out between December 2022 and June 2023, with intensive fieldwork in Rio de Janeiro from March to June 2023), explored sex work, violence, resistance, and practices of care among cis and trans women sex workers.

For the purposes of this article, the two studies were placed in dialogue as they shared overlapping empirical fields and analytical concerns. Both studies followed collaborative approaches [46] and a multi‑sited ethnographic design [47], grounded in situated knowledges [48]. Methodological choices were guided by epistemological, ethical, and political commitments [49]. Drawing on social justice as a “politics of doing” [50], authors approached research as praxis, using qualitative and participatory strategies that could adapt to participants’ needs and foreground their experiential expertise, treating them as co-producers of knowledge.

Together, the two studies allowed to examine how gender-based violence (GBV), substance use, sex work, and harm reduction intersect across different trajectories, with particular attention to gender identity, race, class, territory, and lived experience.

### Participants and recruitment

Participants included trans women and *travesti* engaged in sex work, peer educators, harm reduction workers with and without lived experience, health professionals, and activists. Recruitment was guided by an intersectional approach, ensuring that participants reflected diverse racial, class, and gendered positions within Rio’s trans and sex worker communities. This approach stems from an understanding of the limitations of biomedical framings of suffering [51] and the need to construct counter‑hegemonic readings. Recruitment was carried out through snowball sampling involving community networks, health services, NGOs and activist groups. This ensured the inclusion of both individuals with lived experience of GBV and substance use, and those directly involved in harm reduction practices. As the studies involved marginalised and criminalised contexts, recruitment depended on gradual relationship-building, mediation by trusted community actors, and careful attention to safety and confidentiality.

### Data collection

Data were generated through multiple qualitative and participatory techniques.

In the first study, preliminary network mapping began in October 2018 through conversations and encounters with key actors in trans health, harm reduction, activism, and community-based care in Rio de Janeiro. These included people connected to harm reduction initiatives, trans health projects, a *Centros de Atençao Psicossocial Alcool e Drogas* (CAPSAD—Psychosocial Care Centre for Alcohol and Other Drugs) night outreach service,^3^ community organisations, and activist networks. This mapping informed the selection of two main field sites—which were carried out between 2019 and 2020: one formal care device, the CAPSAD night service, and one informal/community-based care device, a non-governmental organisation supporting trans people. The study also included 10 semi-structured interviews with key actors in formal and informal networks of care and activism, and 4 in-depth life history interviews with trans women and *travestis*, focusing on trajectories of marginalisation, violence, street-based life, access to services, mental health, self-care, and community support.

In the second study, a preliminary mapping phase was conducted between December 2022 and March 2023 to identify key actors, organisations, services, and community initiatives working at the intersections of sex work, harm reduction, gender-based violence, and health in Rio de Janeiro. Through informal exploratory conversations, attendance at community events and meetings, visits to health and social care services, and engagement with activist and harm reduction networks, this phase informed the selection and facilitated access to field sites, while helping establish relationships of trust. Subsequent fieldwork was conducted between March and June 2023 with daily participant observation in two sex work areas and in associated activist collectives, health centres, and NGOs. Data collection also included 18 semi-structured interviews with sex workers, activists, harm reduction workers, and health or social care professionals, and 4 *roda de conversa*^4^—collective conversation circles—involving between 5 and 13 participants each, who had been involved in interviews or participant observation.

Most semi-structured interviews lasted between 45 and 90 min, and involved different participants, although two participants had also been involved in both studies, while conversation circles and life-history interviews lasted between 120 and 180 min.

All interviews and conversation circles were audio-recorded and transcribed with participant consent. Participant observation was documented through fieldnotes and reflexive field diaries. Observations and emerging themes from the field that were not captured in interview transcripts were brought into the conversation circles for collective discussion and reflection. These circles functioned as spaces for dialogue, reflection, and preliminary co-analysis of emerging themes, and also incorporated participatory techniques such as visual mapping of care networks and clay workshops. These creative methodologies were integrated to allow participants to express experiences that might not be easily captured through conventional interviews.

### Data analysis

Data from both studies were brought together and analysed using thematic analysis [52], supported by the Framework Method [53]. The analysis followed three intersecting domains: (1) experiences of GBV and structural violence, (2) substance use and harm, and (3) community-based harm reduction practices.

Coding was conducted independently by members of the research team and subsequently compared and discussed to refine interpretations and resolve discrepancies. Where possible, emerging findings were also discussed with participants during conversation circles and informal feedback exchanges. An intersectional and socio‑anthropological perspective informed the interpretation of the material, allowing comparison across cases, field sites, and forms of data.

Rigour was ensured through multiple strategies: triangulation across interviews, life histories, conversation circles, fieldnotes, and team-based discussion; detailed descriptions of research contexts and participants' trajectories to support transferability; and reflexive field diaries, analytic memos, and coding records to ensure dependability and confirmability [54]. Reflexivity remained central throughout, given the researchers' different positionalities in relation to the field and the reciprocal influence between researcher and research process [49, 55].

### Ethical considerations

Both studies were approved by institutional review boards (Universidade do Estado do Rio de Janeiro and Universitat Rovira i Virgili). Written informed consent was obtained from all participants for interviews, life history and conversation circles. In participant observation and informal ethnographic encounters, consent was treated as an ongoing process through informal feedback exchanges and during conversation circles. To protect anonymity, pseudonyms are used, and potentially identifying details have been removed. Special care was taken to ensure safety and confidentiality given the participants’ exposure to violence, stigma, and criminalisation.

## Results

The ethnographic material revealed four interrelated themes that illuminate how GBV, substance use, and harm reduction intersect in the lives of trans women and *travesti* in Brazil. The results are divided in:

1. Gender-based violence and trajectories of marginalisation:
2. Substance use as coping and as risk:
3. Harm reduction from the margins: community practices of care:
4. Agency, resistance, and re-signification of marginality:

1. Gender-based violence and trajectories of marginalisation

Participants’ life histories underscored the pervasive role of gender‑based violence (GBV) in shaping trajectories of exclusion. Violence was experienced across multiple domains—within families, schools, institutional settings (i.e. health services), and intimate relationships—and was described as both physical and symbolic.

“I am a woman who never bled every month. I bled for a long time, every day, every hour, and every minute for years. For almost 20 years of my life […] I knew that the way I bled was not synonymous with femininity, but rather synonymous with a lot of pain that no one but me could perceive how much I bled. At 14, I was already a woman, I had hair, breasts, my body… This woman had to face the streets at 14, she had to face difficulties […] at 14, I experienced my first rape […] and that rape by four men led to many others, unspeakable, I can't even count them… And violence isn't just physical aggression, it's also verbal or visual. […] But I think it's crazy: a drug dealer who walks down the street, kills, destroys families, and does a lot of things is idolized, but I walk down the street and haven't hurt anyone, and I get booed, and many of us die.” (Bruna,^5^ 35 years old, white, former sex-worker, heterosexual, trans woman)

Many participants recounted being expelled from their homes as adolescents when their gender identity became visible, or being forced out of school due to harassment.

“When my family found out I was transitioning, they kicked me out of the house. They told me I was a filthy, ragged lunatic who deserved nothing and deserved to die. At first, I decided to stop transitioning and stay with them, but even though I started dressing like a man again, my brothers and father beat me every night.” (Gloria, 42 years old, black, harm reduction agent with lived experience, heterosexual, travesti)

“The house pushed me to the street… when the family doesn’t expel you directly, the hostility makes you leave anyway.”(Jessica, 39 years old, Black, advertising student, heterosexual, trans woman…)

Healthcare settings were also described as sites of violence and exclusion, where transphobia and institutional fragility undermined access to care.

“The SUS can be sexualizing and discriminating. […] Some professionals, whether due to transphobia or lack of knowledge, are unable to provide this treatment to trans women at the clinic… She suffers transphobia there, she cannot access care.” (Gloria, 42 years old, black, harm reduction agent with lived experience, heterosexual, travesti)

Participants also recounted violence within sex work itself, due to the impossibility of self-organising of sex workers correlated to the current legal framework^6^ and the struggle to denounce violence due to stigma.

“One day I accepted a job with a client I didn't know […] after some time I told him I had to leave… […] At that moment, he took out a gun, pointed it at my head, and told me I wasn't going anywhere until he decided… […] I kept quiet because in my head I was wrong […] in my mind… what I suffered was part of the risk. I thought, "I'm here doing a job that is wrong, that no one can know I do this, so I suffered this violence and I'll have to bear it alone in silence…” (Cleide, 29 years old, black, former sex-worker, activist, heterosexual, trans woman)

Furthermore some participants highlighted how also the sex work industry could be discriminating against trans people:

“In brothels, travesti stay hidden. When a client arrives, the manager says that there are also travesti there… But there are no brothels exclusively for trans women.” (Gloria, 42 years old, black, harm reduction agent with lived experience, heterosexual, travesti).

“They [cis prostitutes] always had a distant relationship with travestis. […] They never mixed in the places where they work. Usually, travestis are prohibited from entering, so only one or two are allowed in. There is a lot of transphobia in prostitution, right? So it's very complicated.” (Erica, 45 years old, indigenous, sex worker, activist, bisexual, trans woman)

Taken together, these accounts reveal GBV not simply as a series of personal experiences but as a pervasive structure that organises the life chances of trans women and travestis in Brazil. Violence was embedded across families, schools, health services, labour markets, and policing—including within sex work spaces themselves—producing cumulative layers of dispossession and oppression shaped by racism, class inequality, and cisnormativity.

2. Substance use as coping and as risk

Experiences of trauma, sexual violence, and loss were sometimes linked to substance use in participants’ narratives. Drugs and alcohol were described not only as substances but as tools for managing pain, loneliness, and exclusion.

“How not to use? People are poor, Black, rejected by family… drugs make the suffering a little lighter.” (Serena, 37 years old, Black, harm reduction worker, heterosexual, trans woman)

For many, substance use was a way to endure the weight of systemic violence and to create fleeting moments of connection.

“The money I got today was to eat tonight, not for the future… so drugs made me feel less alone”. (Bruna, 35 years old, white, former sex-worker, heterosexual, trans woman)

“I felt alone. Do you know what it's like for people to be close to you because of what you can provide? Working on the street, earning money, and using drugs brought people closer to me. It was a way to have people around, to escape loneliness.” (Gloria, 42 years old, black, harm reduction agent with lived experience, heterosexual, travesti)

For some, alcohol and drugs were also tied to grief and despair, blurring the line between coping and self‑destruction.

“It took me a long time to understand, when I lost my mother, my father… […] Then I discovered alcohol abuse. I would go out to work, but before leaving I would cry and drink…” (Bruna, 35 years old, white, former sex-worker, heterosexual, trans woman)

Substances were also introduced in the context of sex work encounters, with clients offering higher payments in exchange for use. While biomedical frameworks tended to pathologise these practices, participants framed them as inseparable from the broader social suffering generated by GBV and exclusion.

“sex workers use a lot of drugs to keep herself alert and awake, because the more sex she has, the more… well… the more sex, the more money, and as I told you, we get into a vicious cycle, our consciousness changes, we become another person. […] I only slept three hours a night for five years of my life when I was a prostitute, it was a lot of work. I had to work, I had to work out, I had to do prostitution, and I knew it would be for a short time, but that it would be worth it later…” (Cleide, 29 years old, black, former sex-worker, activist, heterosexual, trans woman)

Several participants described substance use as connected to the physical and emotional demands of sex work. Drugs and alcohol were used to stay awake, endure long working hours, manage fear or exhaustion, and respond to the pressure to earn as much as possible within a short period of time. In these accounts, substance use appeared closely linked to working conditions, client expectations, and the absence of effective protections for sex workers.

Alcohol consumption, in particular, was embedded in the economic logic of prostitution, the illegal framework and the lack of policies to safeguard sex workers, as brothel owners pressured sex workers to drink with clients to generate profit.

“Another point that comes up [when I think about health issues] is the labor issue, health doesn't stand alone, right? It has to be political, it has to be incisive, it has to be all policies together, because this problem is now going to be physical? For me, the physical aspect has to do with the lack of legislation, because you are not recognized as a worker. So, to work in certain places there are rules, you work where you want, of course, but in most brothels you have to drink drinks. You can't drink beer. Because every drink you have… Then you have to make the client pay. To boost income, and make them pay for the drink, which is expensive, which is how they make money. So we drink a lot. Prostitutes drink a lot, they drink a lot. […] So for me, I think it's the high consumption of alcohol and drugs, because afterwards I had liver problems, right? Liver. And I'm sure it has a lot to do with the amount of drinking… […] You know? Because many age quickly, because you sleep little, drink a lot, use drugs… for the client. And that's where the problems come in…” (Lisa, 40 years old, black, sex worker, activist, heterosexual, cis woman)

Many explained that drugs entered their lives through clients, who offered more money for encounters involving substances.

“When I discovered prostitution, I thought it would be the only way to survive and get out of the life I was living there. Until I got to the point of discovering drugs. […] But that's what often leads sex workers to drugs, because the price doubles when you have a client who uses drugs. The price doubles. Then that tempts you, tempts the person, right? It tempts you. Then when you realize it, you're already in that situation [drug abuse].” (Sandra, 49 years old, black, harm reduction agent with lived experience, heterosexual, travesti)

Participants also challenged the stigma that associates sex work uniquely with drug use, pointing out that substance use is widespread across society.

“People like to say that many people in prostitution use alcohol or drugs to cope with their work. But not at universities. Guys! I'm here all the time, I have several friends who are academics. I don't use cocaine, for example. All my drugs are legal: beer, whiskey, caipirinhas, coffee, sugar. All my drugs are legal. But how many university professors, students, and administrators use cocaine? An addiction is when you lose control, and that's when something becomes an addiction. […] But looking at prostitution through that lens [of addiction], when it's already a stigmatized, marginalized, and dangerous occupation, “most people in prostitution use drugs,” that's also a lie. Because the vast majority of society uses drugs. And most of the drugs that prostitutes use? Most of the time, it's the clients who bring the drugs. It is the clients who bring them or ask if they know someone from whom they can buy drugs. It is their clients…” (Erica, 45 years old, indigenous, sex worker, activist, bisexual, trans woman)

Finally, participants recognised that substance use could generate new risks, including health complications, heightened exposure to policing, and intensified stigma. This ambivalence—substances as both survival strategies and sources of harm—was a recurring theme. Substance use emerged as a means of survival, connection, and economic gain, but also a site of exploitation, stigma, and marginalisation. Rather than reducing these practices to pathology, participants framed them as embedded in their social conditions and in the economic logics of their work.

3. Harm reduction from the margins: community practices of care

Despite structural barriers and exclusion from formal health systems, participants generated situated harm reduction practices that mitigated risk and fostered solidarity. These practices were rooted in lived experience, collective knowledge, and everyday improvisation. They echoed harm reduction principles but emerged from within informal spaces, shaped by intersectional experiences of gender violence, racism, poverty, and transphobia.

### Peer‑led outreach grounded in lived experience

CAPSAD night teams, often staffed by trans women, *travestis* and sex workers with lived experience, were described as more accessible and trustworthy than traditional clinical care. Their presence not only encouraged trust but also shaped therapeutic decisions. In multiple occasions, psychiatrists and social workers frequently reconsidered treatment plans after peer educators highlighted forms of oppression or alternative interpretations, invisible to biomedical frameworks, highlighting how specific therapies could not be fulfilled in such marginalized contexts.

Furthermore the role of people with lived experience as well as the presence of trans women and *travestis* was perceived as more accessible and trustworthy than traditional clinical care.

“With all respect to the health professionals at the centre, which I think they all do a good job, I do feel a special connection with Gloria… And I trust her… She was on the streets with me, she knows what it’s like, she is not a privileged white girl that learnt from the books… She will never force me to do something that I don’t want to do and she know exactly what I need…” (Sofia, 49 years old, white, sex worker, heterosexual, trans woman)

This credibility was central to engagement, and according to participants was also determined by the embodiment of a “street code” and language.

“When you come from the streets you learn a certain type of language, a certain type of attitude, which you can’t learn in University…” (Gloria, 42 years old, black, harm reduction agent with lived experience, heterosexual, travesti)

### Informal knowledge networks and self-harm reduction

WhatsApp groups functioned as spaces where trans women and *travestis* exchanged advice on hormone use and its interactions with PreP (HIV pre-exposure prophylaxis) and drugs, silicone injections, and safer practices—knowledge otherwise unavailable in formal services.

“We have a WhatsApp group. And there's the “girls' health chat” [trans women and travesti]… this group is ours [trans women and travesti], right? Here, we're done with the psychiatrist who knows everything, right? No way! It becomes a means of communication between LGBTQIA + people and health… We work hard on this issue of putting the doctor in the background… In the group, the conversation flows between us. […] Access to the group is free, everyone is an administrator…[…] We only speak as health workers when something is necessary. To guide or intervene… They are the ones who say how to take hormones, what dosage and which hormone is good for what (breasts, penis, muscles…), the professionals who are there are only there to provide information regarding opportunities, access to services or if there are issues for which they are directly requested…” (Gloria, 42 years old, black, harm reduction agent with lived experience, heterosexual, travesti)

Beyond hormones, sex workers also shared “tricks of the trade” related to substance use and safety. While some of these practices remained deliberately undisclosed, participants emphasised that they were passed down from more experienced colleagues.

Others described private self‑care strategies to manage substance use.

“When I was using drugs and my father was alive, I was able to reconcile it with harm reduction, you know? Without visiting any mental health centers, I would lock myself in my room at home and no one could tell…” (Sandra, 49 years old, black, harm reduction agent with lived experience, heterosexual, travesti)

### Collective care and safe spaces

In the absence or impossibility to access healthcare services due to transphobia or other barriers, participants often reported taking care of one another and improvising harm reduction techniques which could however be impacted by external factors.

“My friend who brought me into the world of sex work had developed a serious substance abuse problem… Gradually, crack had consumed every part of her life, and one day I found her in one of the rooms vomiting blood, and she begged me to help her… I took her to my mother's house for six days and we took care of her… She got clean, but after a while her ex-boyfriend took her back to the “drug tunnel”…” (Sonia, 35 years old, black, sex worker, heterosexual, trans woman)

Harm reduction also took the form of collective defence and vigilance in sex work zones. Participants described intervening when colleagues were attacked or cheated by clients.

“If a customer doesn't pay or hits one of our colleagues, of course we all take action… it's in everyone's interest to have decent working conditions…” (Carla, 25 years old, white, sex worker, bisexual, trans woman)

Others stressed the importance of sharing information about dangerous clients:

“It’s important that we tell at least one of our closest colleagues: ‘Look, that guy stole my money!’… so that other people don’t go through what you went through.” (Nina, 30 years old, sex worker, activist, lesbian, trans woman)

Despite the impossibility to work together in organised context due to current legal policies, on the street, women often worked in pairs, watching over each other during encounters.

“On the street… I liked to be with a colleague… I would tell her when I got in the car, “Look closely at the client's face… look at the license plate… the color of the car…” It may not solve everything, but it discourages the man from doing something bad.” (Raffaela, 33, black, sex worker, trans woman)

This collective protection extended even into hostile institutions. When one of them faced transphobic treatment in a health service, others would accompany her to “armar um caos”—make a mess—and demand accountability.

Yet these networks were deeply ambivalent: solidarity and rivalry were not opposites but co-constitutive features of survival in conditions of structural abandonment. The same precarity that made collective care necessary also fuelled competition and mistrust.

“On the track you are alone… today’s client may find my colleague prettier tomorrow”. (Erica, 45 years old, indigenous, sex worker, activist, bisexual, trans woman)

Care was thus a resource and a fragile practice at once, constantly threatened by structural violence, economic precarity, and the absence of institutional protection.

4. Agency, resistance, and re-signification of marginality

While violence and exclusion structured participants’ lives, the margins were also described as spaces of possibility. Sex work, for many, was not only a survival strategy but also a way to affirm gender identity, exercise autonomy, and access income otherwise denied in the formal labour market.

“So when I was 19, I left home, and since there weren't many services that could support me at the time, I decided to enter the world of prostitution. There I found a new world, not only did men not beat me, but they also paid me to be who I was!” (Sonia, 35 years old, black, sex worker, heterosexual, trans woman)

Several participants framed sex work as a deliberate choice among limited but real options, highlighting the agency involved in deciding how to inhabit their gender identities and sustain themselves.

“When you socialize as a trans woman or travesti, you have two job options: either be a hairdresser or be a prostitute, there isn't much choice! As I had already tried being a hairdresser and didn't like it, I opted for prostitution.” (Fabiane, 45 years old, white, sex worker, bisexual, trans woman)

Others emphasised that sex work offered a space of affirmation compared to the discrimination they faced in formal labour markets.

“Before entering prostitution, I worked as a domestic worker, but I wore men's clothes. When my boss found out I was undergoing hormone treatment, he tried to fire me… I'm already black, poor, and trans. Things are even tougher for me, right? I don't have a great cv, I already lived in a place that was frowned upon, so it's already difficult for you to enter the job market. So how are you going to pay your bills? That's when you get into prostitution, right?” (Gloria, 42 years old, black, harm reduction agent with lived experience, heterosexual, travesti)

Sex work was also narrated as a profession that aligned with political values, offering freedom from the rigidities of formal labour markets.

“I have always seen prostitution as one of the professions that has freed me most from capitalism. It has also given me total freedom to live my life as I want. Prostitution gave me total freedom to travel, to do things at my own pace, to use my time as I wanted. So it was a profession where I chose when I was going to work, I chose the client I was going to see, I had the advantages, the power, the opportunity of always being able to choose, not least because I had a certain body type.” (Erica, 45 years old, indigenous, sex worker, activist, bisexual, trans woman)

Several participants emphasised that prostitution provided freedom and even the basis for chosen families.

“For a travesti in prostitution, the word ‘family’ has a special meaning… the brothel mother, the sisters, the daughters. For those expelled from home or who preferred to leave rather than continue suffering abuse, prostitution represents a space of freedom—a place where you can begin your life, exist as you want, start your transition, and claim your place in the world.” (Gabriela, 53 years old, white, sex worker, activist, lesbian, trans woman)

Importantly, agency was also expressed through trajectories of “recovery” and professional transformation. Some participants who had been sex workers and substance users described how being cared by the CAPSAD opened the possibility of imagining different futures. Through these experiences, they later became harm reduction agents themselves, using their lived experience as a source of credibility and political strength.

“I was once on the other side, using and surviving. But CAPSAD didn’t just treat me—they involved me. They showed me I could be part of the team. Today I am a harm reduction agent, and when I talk to someone on the street, they believe me because I’ve been there too.” (Sandra, 49 years old, black, harm reduction agent with lived experience, heterosexual, travesti)

These trajectories were recurrent across both studies. Participants who had navigated sex work, substance use, and structural violence described how encounters with peer-led care—and with others who shared their experience—opened possibilities that formal services had rarely offered: being seen without judgement, accessing information without stigma, and imagining futures that had previously felt foreclosed. Several had gone on to become harm reduction workers, peer educators, or activists, carrying their lived experience into their professional roles as both a tool and a form of authority.

What emerged, across all four themes, was a portrait of subjectivities who were simultaneously targets of multiple, intersecting forms of violence and active producers of knowledge, care, and resistance—making harm reduction not only a health practice but a collective act of survival.

## Discussion

This study demonstrates how gender‑based violence, substance use, and harm reduction intersect in the lives of trans women and travestis in Brazil, contributing to a growing body of scholarship that calls for intersectional and community-centred approaches to harm reduction [6, 11, 41, 43, 56]. By foregrounding participants' narratives, the analysis confirms that GBV operates not as a series of isolated incidents but as a structural force embedded across families, schools, health services, labour markets, and policing—shaping substance use trajectories in ways that biomedical frameworks alone cannot capture [8, 9, 41, 43]. At the same time, the findings extend existing literature by showing how women actively navigated these conditions: developing situated harm reduction practices, re‑signifying sex work as a space of affirmation, and transforming lived experience into professional and political expertise. In doing so, the study advances the argument that the margins are not only sites of exclusion but also of creativity, solidarity, and resistance [20, 45]—and that harm reduction, to fulfil its emancipatory potential, must be understood as a political practice of care rooted in these realities.

### Intersectionality and structural violence

Participants' narratives made visible how GBV was enacted and institutionalised across the very spaces meant to provide protection and care. Expulsion from families, harassment in schools, and denial of care in health services were recurrent. Being excluded by family members, forced to "de-transition," or subjected to transphobic treatments in the SUS were not exceptional experiences but structural ones—shaping participants' trajectories from the earliest stages of their lives.

The legal ambiguity surrounding sex work in Brazil further intensified these dynamics. Although prostitution is recognised as a profession, the criminalisation of third parties and organised spaces effectively pushes sex work into illegality and precarity, leaving workers without labour protections or institutional recourse [40, 57]. Beyond the legal framework, stigma operated as a powerful force of de-subjectivation: it prevented participants from recognising themselves as workers with rights, from naming the violence they experienced as such, and from collectively organising to claim those rights. Cleide's account of being held at gunpoint by a client—and her sense that she had to "bear it alone in silence"—illustrates precisely this double bind: criminalisation and stigma worked together to foreclose both legal recourse and political agency. Similarly, participants highlighted how transphobia within the sex industry compounded exclusion: brothel managers hiding travestis from clients, or limiting their presence to "one or two," reinforced hierarchies even within marginalised economies.

These experiences cannot be reduced to single axes of oppression but emerge from the convergence of gender identity, race, class, territory, and sexuality [4]. The findings extend Brazilian public health scholarship on how Blackness, poverty, and drug use intersect to produce vulnerability [13, 35], while also inviting a revisiting of vulnerability itself—not as a fixed attribute of individuals but as a dynamic process produced at the intersection of personal trajectories, institutional practices, and broader social conditions [58].

The life histories collected here illustrate recurrent *itinerários de vulnerabilização*—in which structural processes continually generate situations of social humiliation, exclusion, and premature death [39]—not as the product of isolated "risk factors" but as the historical and political production of health and illness through intersecting systems of exploitation, patriarchy, racism, and cisnormativity [59]. These trajectories point to the operation of structural violence [7], where systemic inequalities reproduce vulnerability while simultaneously obscuring responsibility—and, at their most extreme, to what Mbembe terms necropolitics [60]: the power to decide who may live and who must die.

Participants’ testimonies of being denied care in health services, exposed to police violence, or left unprotected in sex work zones illustrate how trans women and *travestis* (especially if non-white) are positioned as populations whose suffering is normalised and whose deaths are rendered socially acceptable,—a dynamic reflected in the rising numbers of trans and sex worker killings in Brazil [34].

By situating participants' experiences within intersectionality, the social determination of health, and necropolitics, this study underscores that GBV is not only a matter of interpersonal violence but a structural determinant of health inequities and premature death—one that falls disproportionately on Black and Indigenous trans women and travestis, whose exposure to violence, criminalisation, and institutional abandonment is compounded by racism and colonial legacies [13]. It points to the urgent need for health systems and public policy to move beyond biomedical framings of "risk" and address the political and economic conditions—including the legal ambiguity of sex work, pervasive transphobia, and structural racism—that produce vulnerability in the first place, and that harm reduction, to fulfil its emancipatory potential, must therefore address.

### Substance use beyond pathology

Despite recognising the health risks and heightened exposure to violence and policing correlated with substance use, our results question biomedical approaches that pathologise it among trans women, travestis and sex workers. Rather than an individual pathology or moral failure, participants interpreted drug and alcohol use as inseparable from trauma, loneliness, and survival under conditions of GBV [3, 7, 51].

The findings further show that substance use cannot be separated from the labour conditions of sex work. Clients introduced substances into encounters, often doubling payment but increasing risk, while participants also described using drugs and alcohol to endure exhaustion, remain alert, manage fear, and maximise income under precarious and unsafe working conditions. These dynamics are inseparable from the broader commodification of sex workers' bodies and lives under capitalism: the absence of labour recognition, compounded by stigma, dissolved the boundary between work and life, rendering the body itself a site of extraction and exhaustion [40] Substance use must therefore be understood within a broader economy of survival, shaped by gender-based violence, labour insecurity, criminalisation, and the lack of protections for sex workers [7, 51]—and harm reduction interventions must be responsive to these realities rather than punitive.

The use of psychopathological categories to name and contain participants' relationship with drugs often failed to capture the complexity of their suffering, reducing substance use to “psychic suffering” while obscuring its structural and colonial dimensions of trauma. These trajectories of pathologisation can be traced back to nosological discourses that have historically claimed stability and authority while relying on sexist stereotypes to define trans populations [61], and to colonial projects that have always relied on the dehumanisation of certain bodies [62]—relegating them, in Fanon's terms, to the “zone of non-being” [63]. Yet these same categories—“prostitution,” “drug use,” historically attached to street populations and travestis—were not only markers of stigma but also terrains from which alternative forms of knowledge and solidarity emerged.

The intersection of multiple oppressions produced unique vulnerabilities, while also creating conditions for innovative harm reduction practices. Participants described WhatsApp groups where they exchanged information on hormones and safer drug use, peer‑led outreach that drew credibility from lived experience, and improvised clinics that filled gaps left by the SUS. These practices exemplify the principles of popular education, where experiential knowledge circulated and solidarity was enacted [64]. What Clarindo et al. call “saberes da putaria”—sex‑work knowledges blending humour, improvisation, and embodied expertise—were not informal curiosities but counter‑hegemonic forms of health knowledge [65].

At the same time, care was deeply ambivalent. Solidarity was constantly undermined by rivalry, competition for clients, and capitalist pressures, resonating with Mol’s distinction between the logic of care (relational, collective, situated) and the logic of choice (individualised, market‑driven) [66]. In the sex work economy, care practices were simultaneously protective and fragile. Still, they can be read as forms of mutual aid in precarity [45]: sex workers watching each other’s backs on the street, sharing information about violent clients, or collectively confronting transphobic health professionals. Harm reduction here was not only about safer substance use but about reclaiming dignity and survival in hostile environments.

Our findings show that peer-led harm reduction operates as a situated human rights practice. For participants, reducing harm meant more than reducing drug-related risks: it involved creating access to care where institutions were absent or hostile, building trust where discrimination had shaped previous encounters with services, and protecting life in contexts marked by gender-based violence, racism, poverty, sex work stigma, and transphobia [10, 11]. In this sense, peer-led harm reduction gave practical form to the right to health, dignity, non-discrimination, bodily autonomy, and freedom from violence [11, 42]. It also challenged models of care centred on abstinence, institutionalisation, or moral correction, replacing them with relational, territorial, and community-based practices of care [41, 43].

Importantly, this does not mean opposing abstinence as one possible outcome, but rather refusing abstinence as the only legitimate goal of care, especially when it is imposed through moralising, institutionalising, or punitive models [41]. Peer-led harm reduction therefore challenged models of care centred on abstinence, institutionalisation, or moral correction, replacing them with relational, territorial, and community-based practices of care [41, 43].

### Marginality as possibility

At the same time, our data underline that marginality is not only a site of harm but also of possibility. In line with hooks [20], participants re‑signified their positions of abjection, developing alternative forms of knowledge and practice. Peer‑led harm reduction initiatives, WhatsApp groups, and the “saberes da putaria” [65] exemplify what Haraway terms situated knowledges [48]: experiential expertise that challenges biomedical authority and generates counter-hegemonic forms of care. These practices allowed participants to mitigate risks, share resources, and build chosen families in contexts of exclusion.

For many participants, “being on the street” was simultaneously experienced as violence and fear, but also as another way of inhabiting the world [67]—a form of agency [68] when the home represented abuse and exclusion. Agency here was not simply opposition to norms, but the invention of alternative spaces and subjectivities. Sex work itself was rarely narrated as a “last resort” [57, 69]; instead, it appeared as a resource for self‑determination when the state failed or responded violently, allowing professionals to “work for themselves,” accumulate capital for personal projects, and counter economic exclusion and cultural injustice of a patriarchal labour market [40]. This recalls Kilomba’s necessity of going beyond opposition, fostering the creation of new places for those pushed to the margins to resist colonial, patriarchal, and racist projects [62], while Mombaça underscores the need to escape the paths that push trans and *travestis* toward death [70].

These trajectories of re-signification were also visible in participants' professional lives. Several had moved from experiences of sex work, substance use, and structural violence into roles as harm reduction workers, peer educators, and activists—carrying their lived experience as both a tool and a form of authority. This movement from being cared for to becoming carers themselves challenged biomedical hierarchies and embodied what Favero calls the "de-cisgendering" of health knowledge [61]: the claim that trans women and travestis are not only objects of care but producers of it. Lived experience, in this sense, functioned not as a deficit but as a counter-hegemonic form of expertise. Becoming harm reduction workers while drawing on lived experience, participants transformed histories of exclusion into professional authority—claiming a form of expertise that biomedical training alone cannot produce.

By inhabiting these “limit situations,” participants re‑subjected themselves from the margins, liberating themselves from structural violence and reconstructing new forms of existence. Here, contradictions became visible, dominant orders were challenged, and possibilities for refusal and reinvention emerged. Marginality, in this sense, was re‑signified as a site of agency, solidarity, and re‑existence—a place where stigma was transformed into freedom, chosen kinship, and political imagination.

Recognising peer-led harm reduction as a human rights practice means recognising trans women and travestis not only as populations exposed to vulnerability, but also as producers of care, knowledge, protection, and political claims [42, 71]. In contexts where punitive drug policies, gender-based violence, racism, and transphobia restrict access to rights, these community practices become ways of making the right to health, dignity, and life materially possible [11, 42].

### Implications for harm reduction and policy

These findings have several implications for the future of harm reduction and public health policy. First, harm reduction must explicitly integrate gender‑based violence (GBV) as a central concern, recognising its role in shaping substance use trajectories and health outcomes. Critical public health scholarship has long argued that violence is not peripheral but a structural determinant of health. Trauma‑informed and survivor‑centred approaches are therefore essential to ensure that harm reduction services respond to the realities of trans women, *travestis* and sex workers rather than reproducing exclusions.

Second, policies should support and integrate and recognise people with lived experience and community‑led harm reduction practices—without undermining their autonomy or subordinating them to institutional agendas. Evidence from harm reduction research shows that peer‑driven initiatives are more effective in reaching criminalised populations and building trust [56]. However, institutional recognition carries the risk of co-optation: the incorporation of community practices into formal systems can strip them of their political character, reduce peer workers to cheap labour, and reproduce the hierarchies they were designed to challenge. Funding and recognition must therefore be accompanied by genuine power-sharing, adequate remuneration, and structural protections that safeguard the independence and transformative potential of these practices.

Third, services must adopt intersectional and anti-racist approaches that address not only substance use but also the overlapping effects of gender identity, race, poverty, and stigma. While intersectionality has been increasingly mobilised in public health to highlight how health inequities emerge from converging systems of oppression, its implementation remains far from accomplished [5, 43]. In the Brazilian context, this requires integrating anti-racist strategies into health services as a structural—not supplementary—commitment: connecting with Black and Indigenous heritage groups and community movements, ensuring trans-affirming care that includes access to hormones, PrEP, and broader sexual and reproductive health services, and creating anti-racist committees within psychosocial care networks [72]. Anti-racist care cannot be constructed "for them, without them"—it requires the active protagonism of users, workers, and social movements, and must be treated as indispensable to any harm reduction agenda [43, 72].

Fourth, decriminalisation of sex work is a critical step. As demonstrated, criminalisation increases vulnerability to violence, limits access to health services, and undermines collective organising [1, 73, 74]. Decriminalisation, combined with labour protections, would allow sex workers to negotiate safer conditions, reduce client‑driven coercion into substance use, and strengthen solidarity networks.

Finally, harm reduction must be reframed as a political practice of resistance to patriarchy and coloniality. This includes comprehensive gender and sexuality education in schools, destigmatisation campaigns for dissident genders and sexualities, and the inclusion of people with lived experience in the design, delivery, and evaluation of services. As Spade argues, mutual aid and community‑based care are not supplements to formal health systems but essential strategies for survival in contexts of precarity [45].

Taken together, these implications call for a shift from narrow biomedical framings of harm reduction toward a critical public health agenda that recognises GBV, racism, transphobia, and criminalisation as structural determinants of health. This shift is not only a matter of policy design but of human rights: the right to health, to dignity, to bodily autonomy, and to freedom from violence are not abstract principles but material claims that harm reduction—when practised in its fullest, most political sense—can help make possible [11, 42]. Only by addressing these intersecting forms of exclusion can harm reduction fulfill its promise of dignity, safety, and self‑determination for trans women and *travestis*.

### Limitations

This research offers an intersectional and ethnographically grounded perspective on harm reduction that centres GBV as a structural determinant of health. By foregrounding trans women and travestis as producers of knowledge and care—rather than merely as populations at risk—it advances critical public health frameworks that move beyond biomedical and pathologising approaches to trans identities and substance use. However, this study has limitations. Firstly, it focuses exclusively on trans women and *travestis*, which highlights the need for future research that includes other subjectivities. Secondly, while ethnographic depth was achieved, the absence of a fully co‑participatory writing process limited the extent to which interlocutors could shape the final text. Finally, the findings are situated in Brazil and may not be generalisable to other contexts. These constraints underscore the importance of further participatory and comparative studies to expand the scope of harm reduction research.

## Conclusion

This study examined how gender‑based violence and substance use intersect in the lives of trans women and travestis in Brazil, and how community harm reduction practices emerge in response. Drawing on two ethnographic inquiries placed in dialogue, and analysed through an intersectional and socio‑anthropological lens, the findings show that GBV and psychoactive substance use are inseparable from interpersonal, institutional, and structural violence—producing recurrent *itinerários de vulnerabilização* [39] that limit participants to precarious survival strategies while simultaneously generating conditions for resistance and care.

At the same time, the findings demonstrate that marginality is not only a site of harm but also of possibility. Amid violence, suffering, and pain, women mobilised formal and informal solidarity networks, community care, and shared resources to enact harm reduction practices rooted in situated knowledges [48]—from WhatsApp groups to peer‑led outreach and the saberes da putaria [65]. These practices illustrate how trans women and travestis transform spaces of exclusion into arenas of resistance, care, and re‑existence, refusing to be reduced to their suffering.

By centering GBV within harm reduction, this research advances critical public health debates that have often overlooked violence as a structural determinant of health. It contributes to global harm reduction scholarship by offering a perspective rooted in the Global South, where the harms to be reduced are inseparable from histories of colonialism, racism, patriarchy, and neoliberal precarity—and where community-based practices of care constitute, in themselves, a form of political claim-making. In the Brazilian context specifically, the anti-racist struggle must be integrated into harm reduction not as a supplementary concern but as a structural one.

Ultimately, this study underscores that harm reduction cannot be understood apart from the structural conditions that produce the harms it seeks to address. By centring the lives, knowledges, and practices of trans women and travestis in Brazil, it advances a conception of harm reduction that is at once intersectional, decolonial, and politically grounded—one that recognises GBV not as peripheral to substance use but as constitutive of it, and that insists on community-generated knowledge as a legitimate and necessary foundation for public health practice and human rights approaches. In doing so, it argues that the everyday practices of trans women and *travestis*—of care, solidarity, and survival—constitute, in themselves, a form of political claim-making that harm reduction scholarship can no longer afford to ignore.

## Data Availability

No datasets were generated or analysed during the current study.

## Footnotes

1 In the present article, the authors have chosen not to translate the term *travesti*. The word carries a decolonial perspective that cannot be adequately rendered in English: it refers to subjectivities that disrupt binary gender categories, resist the reductionist and pathologizing gaze of biomedicine, and challenge capitalist, patriarchal, and colonial forms of violence. Beyond describing gender variance, *travesti* names a political identity and a collective practice of survival and resistance, rooted in Latin American histories of marginalization and activism. Indigenous trans and *travesti* activists emphasize that the term revives plural, non‑binary identities embedded in ancestral traditions and knowledges that were erased by the gender binary imposed through Catholicism during colonization. To translate it would risk erasing these specific genealogies and struggles; leaving it in its original form acknowledges its epistemic and political force.

2 We use the term trans to encompass all subjectivities who define themselves as such, not in the sense of reifying biomedical categories or the biomedical management of subjectivities, but rather to refer to those who transgress the binary sex-gender system [44]. In the Brazilian context, we also recognise the category of *travesti*, which does not map neatly onto English understandings of “trans woman.” *Travesti* historically and politically articulate a gendered existence that challenges both cisnormativity and biomedical classifications, rooted in specific cultural, racial, and classed experiences [44] By including both trans and *travesti*, we aim to foreground local epistemologies and resist the universalisation of biomedical or Western categories of gender.

3 A service that is part of the Psychosocial Care Network within the Brazilian public health system, aimed at people with "serious, severe, and persistent suffering" resulting, in this case, from the "abusive and harmful use of alcohol and other drugs". Regulated, in the context of the Brazilian Psychiatric Reform, by Law 10.216/2001 and Ordinances 336/2002 and 3088/2011.

4 *Rodas de conversa* (literally "conversation circles") are a participatory methodology rooted in Freire's pedagogy of the oppressed [64]. Drawing on the principles of dialogue and critical consciousness (*conscientização*), they are designed as collective spaces in which participants recognise their own subjectivity, develop a shared sense of agency, and engage in mutual political formation [40]. Unlike conventional focus groups, *rodas de conversa* are not researcher-led: participants take command of the conversational flow, centering what they most want to say and fostering an exchange of perspectives that generates both awareness and the legitimation of lived experience [55].

5 This is a fictitious name chosen by this woman to present herself in this work. The self-definitions of race, gender, sexuality, and generation have been kept as they were spoken by the people themselves. Ellipses are used to indicate that these are multiple people/existences, far beyond any classificatory categories.

6 In Brazil, prostitution is recognised as a profession (Occupation Code 5198-05, Ministry of Labour, 2002) and is not in itself illegal. However, all activities surrounding it—including the involvement of third parties and the maintenance of spaces dedicated to prostitution—are criminalised under Title VI of the 1940 Penal Code (Decreto-Lei nº 2.848/1940), which deliberately conflates prostitution with sexual exploitation, enabling arbitrary enforcement and effectively pushing sex work into conditions of illegality and precarity.
